# Investigating the Use of a Serious Game to Improve Opioid Safety Awareness Among Adolescents: Quantitative Study

**DOI:** 10.2196/33975

**Published:** 2021-12-23

**Authors:** Olufunmilola Abraham, Claire Rosenberger, Kathleen Tierney, Jen Birstler

**Affiliations:** 1 Social and Administrative Sciences Division School of Pharmacy University of Wisconsin–Madison Madison, WI United States; 2 Biostatistics and Medical Informatics School of Medicine and Public Health University of Wisconsin–Madison Madison, WI United States

**Keywords:** opioids, adolescents, opioid safety, medication safety, opioid knowledge, serious games, naloxone, Narcan, youth, technology, safety, gaming, addiction, drug, young adult, teenager, survey, awareness

## Abstract

**Background:**

The misuse of opioid medications among adolescents is a serious problem in the United States. Serious games (SGs) are a novel way to promote the safe and responsible management of opioid medications among adolescents, thereby reducing the number of adolescent opioid misuse cases reported annually.

**Objective:**

This study aimed to examine the effect of the SG MedSMART: Adventures in PharmaCity on adolescents’ opioid safety knowledge, awareness, behavioral intent, and self-efficacy.

**Methods:**

A nationally representative sample of adolescents aged 12 to 18 years were recruited online through Qualtrics panels from October to November 2020. Data collection consisted of a pregame survey, 30 minutes of gameplay, and a postgame survey. The pregame and postgame surveys included 66 items examining the participants’ baseline opioid knowledge, safety, use, and demographic information. The postgame survey had 25 additional questions regarding the MedSMART game. Gameplay scenarios included 5 levels intended to mimic adolescents’ daily life while educating the players about appropriate opioid storage and disposable practices, negative consequences of sharing opioid medications, and the use of Narcan. Survey questions were divided into 10 categories to represent key concepts and were summarized into concept scores. Differences in concept scores were described by overall mean (SD) when stratified by gender, race, school, grade, and age. Differences of change in concept score were analyzed using the Kruskal-Wallis and correlation tests.

**Results:**

A total of 117 responses were analyzed. The results showed significant improvement on all concept scores except for Narcan knowledge (mean change -0.04, SD 0.29; *P*=.60) and safe storage (mean change 0.03, SD 0.20; *P*=.09). Female participants had greater improvement than males for safe disposal (female mean 0.12, SD 0.25 vs male mean 0.04, SD 0.17; *P*=.05). Male participants had higher improvement than female participants for misuse behavior (female mean 0.05, SD 0.28 vs male mean 0.14, SD 0.27; *P*=.04). Perceived knowledge for participants who had non-White or Hispanic racial backgrounds had higher improvement than for non-Hispanic White participants (non-White mean 1.10, SD 1.06 vs White mean 0.75, SD 0.91; *P*=.03). Older grades were associated with greater improvement in opioid knowledge (correlation coefficient -0.23, 95% CI -0.40 to -0.05; *P*=.01). There were 28 (23.9%) participants who played all 5 levels of the game and had better improvement in opioid use self-efficacy.

**Conclusions:**

Our findings suggest MedSMART: Adventures in PharmaCity can be used as an effective tool to educate adolescents on the safe and responsible use of prescribed opioid medications. Future testing of the effectiveness of this SG should involve a randomized controlled trial. Additionally, the feasibility of implementing and disseminating MedSMART: Adventures in PharmaCity in schools and health care settings such as adolescent health or primary care clinics, emergency departments, and pharmacies should be investigated.

## Introduction

Opioid overdose mortality has increased dramatically in the United States. Over the past 2 decades (1999 to 2019), nearly 500,000 deaths from opioid-related overdoses have been reported [1]. Moreover, the growth trend for opioid-related deaths continues to increase over time. Opioid-involved overdose deaths increased by approximately 136%, from 21,088 deaths in 2010 to 49,860 in 2019—over 6 times the number of opioid-related overdose deaths in 1999 [2]. Overdose fatalities are largely driven by heroin use, misuse of synthetic opioids such as fentanyl, and pain relievers available legally by prescription, including oxycodone (OxyContin, Percocet, and Roxicodone), hydrocodone (Vicodin), codeine, morphine, and tramadol (Ultram) [3]. Due to the considerable increase in opioid overdoses, the opioid epidemic has been declared a national public health crisis in the United States [4].

The opioid epidemic affects persons of all ages, genders, and racial and ethnic groups, with 7.6% of adolescents reporting opioid misuse in 2019 [4-6]. Although the reasons for adolescent opioid misuse are complex and vary from person to person, the existing pain management prescribing practices and the lack of guidelines for pain management in children are contributing factors for opioid misuse [7-9]. Currently, the Centers for Disease Control and Prevention does not have guidelines or recommendations for pain management in children, nor does it provide a clear definition for opioid misuse and safety [7,10]. Studies have found 64% of clinicians do not have a standardized protocol for prescribing pain management medications to adolescents, less than half of pediatric providers screen their adolescent patients for substance use, and only 30% offer an intervention, which is often short-lived [8,9]. Furthermore, the most recent data on the US pediatric opioid prescribing practices show almost half of pediatric opioid prescriptions were categorized as high-risk [11].

Several studies have demonstrated that adolescents have inadequate knowledge and understanding about opioid use and safety [12-14]. These studies suggest that adolescents are well-informed on how to use prescription opioids; however, they are underinformed of the addictive potential of opioids, the risks of overdose, and the availability of naloxone (Narcan, Emergent Operations Ireland) to reverse an opioid overdose [15,16]. Additionally, many adolescents are not able to correctly identify which medications are opioids and which are not [13]. It is important to educate adolescents on the safe, appropriate, and responsible management of opioid medications because they are in a developmental stage where they can learn about and implement healthy lifestyle behaviors. Research shows adolescents can be taught to avoid harmful health-related behaviors, especially if they are provided with evidence that correlates dangerous behaviors to potentially dangerous outcomes [17]. Therefore, beginning opioid safety communication and education at an early age is critical for curbing the opioid epidemic, decreasing opioid misuse among young people, and preventing future opioid-related deaths.

The use of serious games (SGs) is a promising approach for promoting proper opioid use and safety among adolescents [14,18-21]. SGs are video games designed not only for entertainment, but to educate persons on a specific topic or topics, change an attitude or behavior, or create awareness of a certain issue [22]. The global SG market was valued at US $2731 million in 2016 and is expected to reach US $9167 million by 2023 based on the games’ desirability and ability to improve learning outcomes [23]. Moreover, 70% of people under the age of 18 regularly play video games in the United States [23]. Due to the significant number of adolescents who play video games, and the international acceptance of these games, utilizing SGs to educate adolescents on opioid use and safety is highly feasible.

Many SGs have had success in achieving their health-related educational purposes. The SG Aislados teaches adolescents skills to prevent drug addiction, sexist behavior, and other risk-related behaviors [22]. The game has been shown to improve adolescents’ attitudes and change their behaviors [22]. Additionally, Recovery Warrior 2.0, a motion-activated video game prototype, which targets relapse prevention for adolescents, has preliminary data indicating that an SG for addiction recovery appears to be possible and appropriate for adolescents [24]. Although several interventions have shown promise in improving adolescents’ health-related behaviors, there are limited studies that have examined the effect of an SG on adolescent’s opioid-safety awareness [20]. This study aimed to investigate the use of the SG, MedSMART: Adventures in PharmaCity in improving adolescents’ opioid safety knowledge, awareness, behavioral intent, and self-efficacy.

## Methods

### Survey Design

Data were collected through a pregame survey, 30 minutes of gameplay, and a postgame survey; a common method to evaluate SGs [25]. Pregame and postgame opioid-related survey questions were adapted from various validated surveys and scales or created by the investigators [26]. Questions from a Wisconsin statewide survey collecting perceptions, awareness, and use of prescription medications in Wisconsin residents were adapted for adolescent use and assessed the participants’ knowledge of safe prescription opioid storage and disposal [14,27]. Questions assessing self-efficacy of prescription opioid use and other learning objectives from the SG were adapted from the MUSE (Medication Understanding and Use Self-Efficacy) scale and a survey assessing workplace safety and health knowledge in adolescents [28,29]. Questions measuring participants’ knowledge about naloxone (Narcan) were adapted from the Maryland Opioid Survey Summary Report [30]. Attention check questions were included in the pregame and postgame surveys to ensure the participants were thoroughly reading survey questions and to prevent straight lining. All surveys were hosted online through Qualtrics (Qualtrics LLC), and the data collected were securely stored through Qualtrics. Surveys were reviewed by Qualtrics staff and the study team to ensure functionality prior to distribution. This study was approved by the Institutional Review Board of University of Wisconsin-Madison.

### Pregame Survey

The pregame survey consisted of 66 nonrandomized items, and 59 of the items were used to examine the participants’ baseline opioid knowledge, knowledge of safety, disposal and misuse, and the perceived effect an SG may have on opioid safety awareness. These 59 items consisted of questions with “Yes,” “No,” and “Don’t know” as answers or a 5-point Likert scale. Two items were also used as attention check questions to prevent straight lining by asking the participants to select a specific option within the 5-point Likert scale. Five demographics questions were asked to assess the participants’ characteristics, including race and ethnicity, grade in school, age, gender, and the number of children under the age of 18 living in their household. The online survey consisted of 12 pages with up to 10 items on each page. The pregame survey questions are included in Multimedia Appendix 1.

### Postgame Survey

The postgame survey consisted of the same 59-item baseline opioid-related questions in the pregame survey and 2 attention check questions, as well as 25 additional questions asking about the participants’ perspectives on the MedSMART game. The postgame survey included a question asking the participants if they had ever been prescribed opioids by a doctor, and questions asking them to list the opioids they had been prescribed and any prescribed medications they perceived might be opioids. Additional personal health information was not collected in the survey per Qualtrics guidelines for surveys recruiting participants through their research panels. Response options included 5-point Likert scale questions, multiple choice questions, free response questions, and “yes,” “no,” and “unsure” answers. The online survey consisted of 17 pages with up to 10 items on each page, and the items were not randomized. The postgame survey is included in Multimedia Appendix 2.

### Intervention

The SG MedSMART: Adventures in PharmaCity was designed to educate adolescents about safe opioid use and management as well as enhancing the players' ability to make informed decisions about proper opioid use in real life. MedSMART: Adventures in PharmaCity was developed over the course of several months by a team of researchers, health care personnel, and game design experts [18]. The key elements of the game were defined, including goals, levels, mechanics, game flow, story, and characters. The game was only playable on a computer or tablet with keyboard access and had simple commands (all player actions were made using keys “A,” “D,” “W,” and spacebar). The game engine was built using Unity (Unity Technologies). The situations presented in the game were designed to be similar to scenarios adolescents may encounter in real life. The game provided immediate feedback to the player if they made a wrong decision, and the player could only progress in the game if they made the right decision and followed the correct sequence in the story line. After the prototype was built, the game was piloted with adolescents and pharmacy students to examine whether they liked the game, understood how to use it, and could navigate throughout the game [18,21]. Their feedback was incorporated into the final game design used in this study.

**Figure 1:**
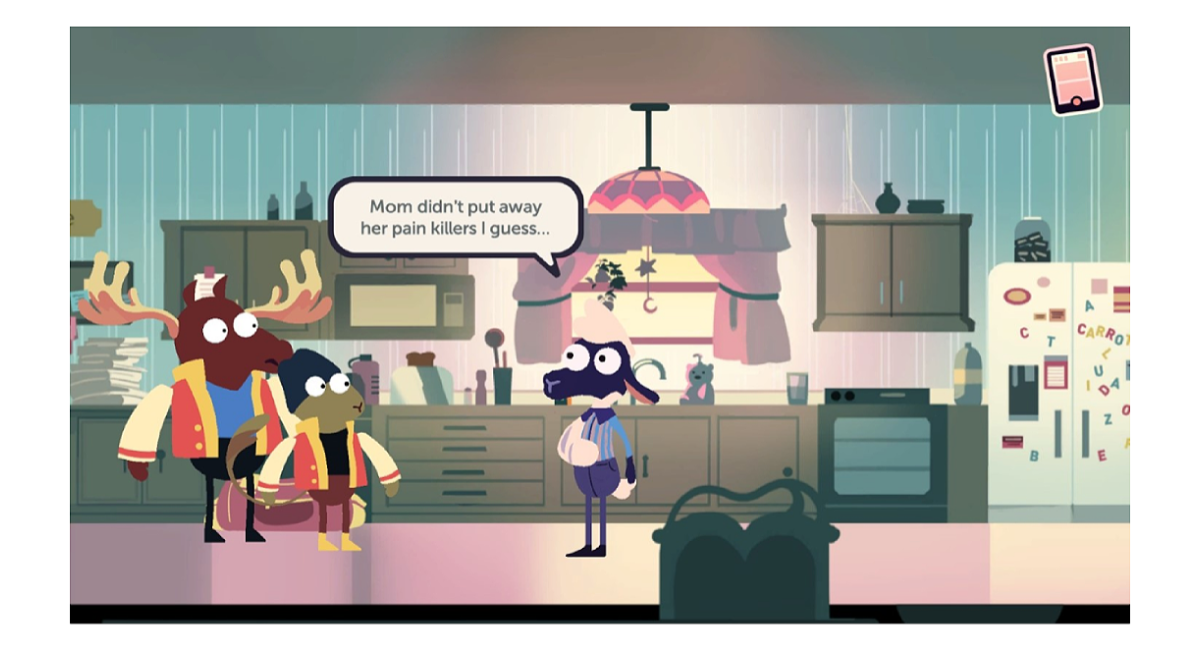
Screenshot from level 1, A Quiet Sunday Afternoon.

**Figure 2:**
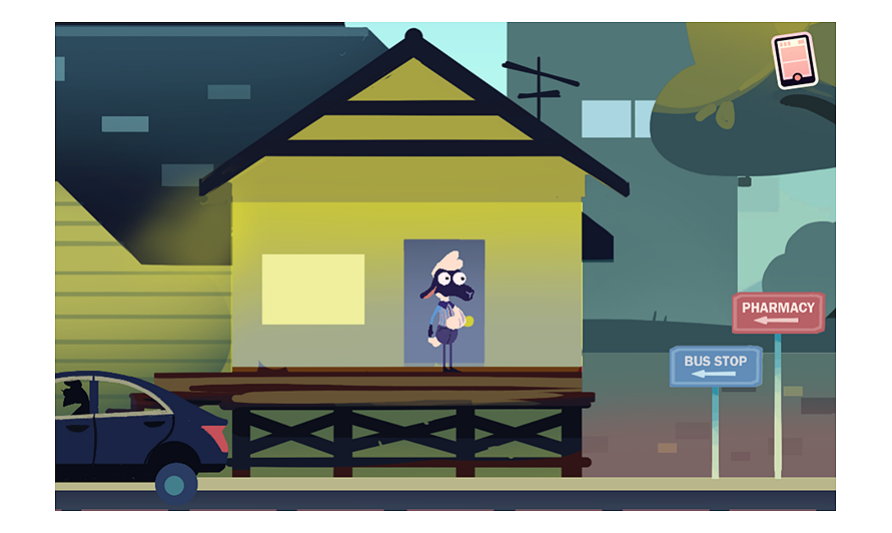
Screenshot from level 2, Monday Morning Bus Ride.

**Figure 3:**
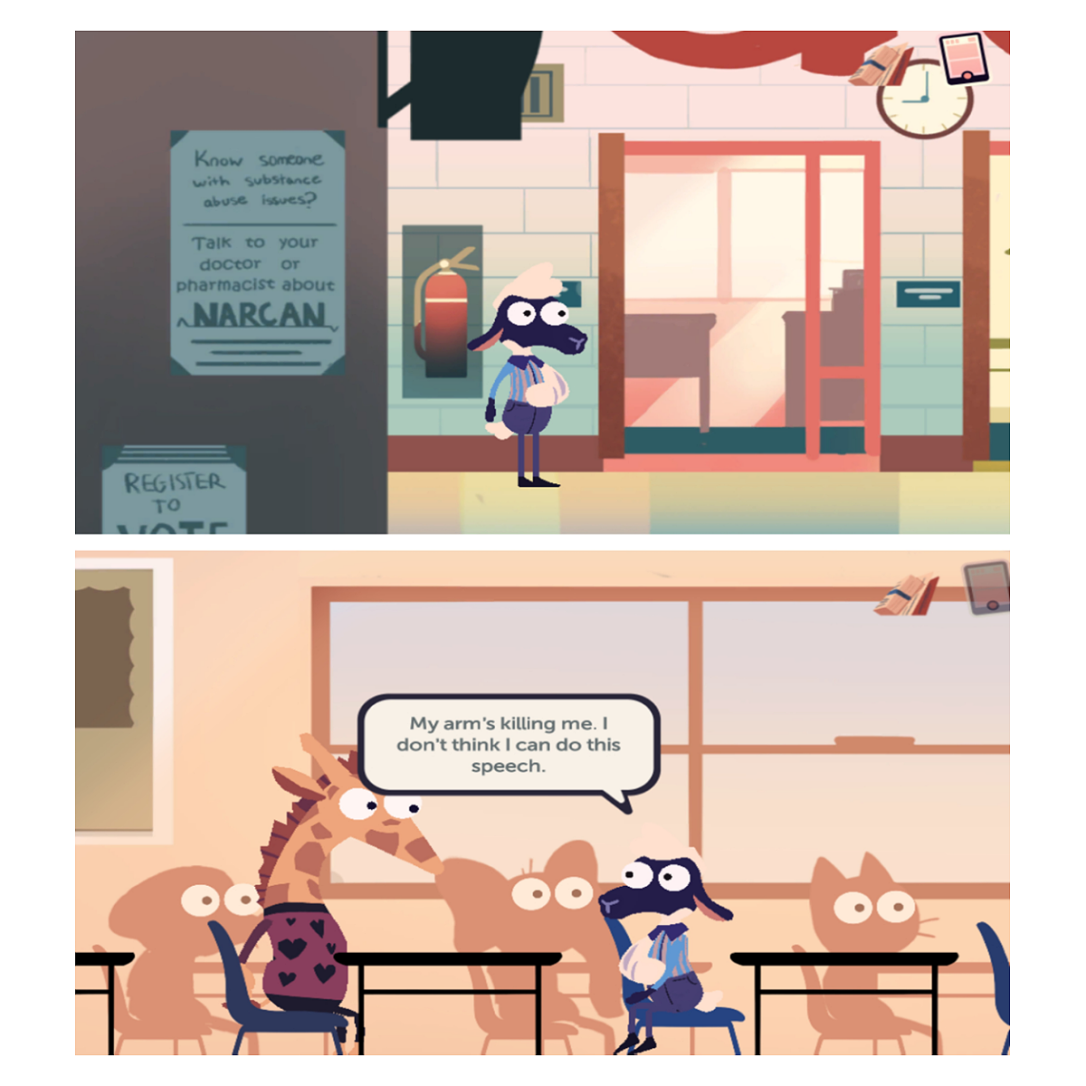
Screenshots from level 3, A Persuasive Speech at School.

**Figure 4:**
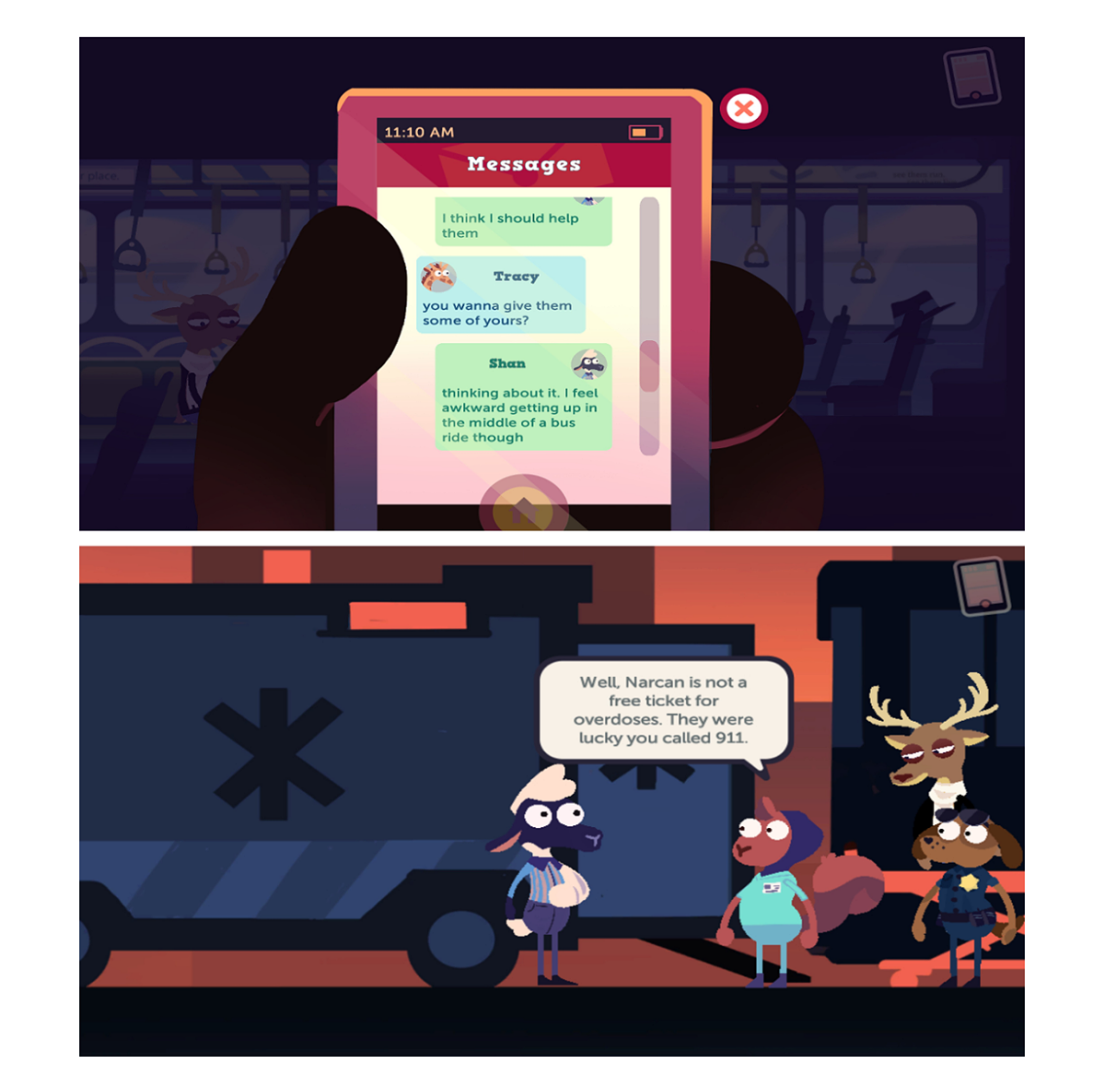
Screenshots from level 4, Bus Ride Home.

**Figure 5:**
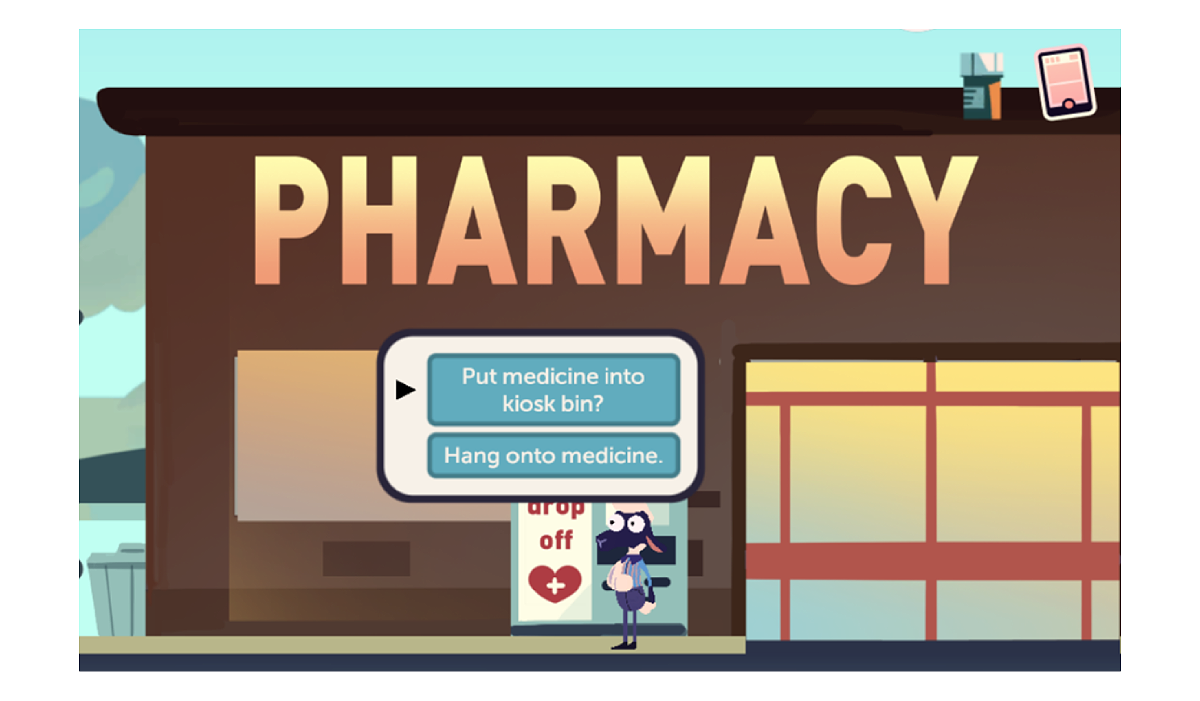
Screenshot from level 5, Last Minute Chore.

MedSMART: Adventures in PharmaCity is a task-guided game divided into 5 levels. Participants play as “Shan,” the anthropomorphized sheep, who has been prescribed opioids after breaking their arm. The player’s objective is to guide Shan to make the right choices regarding the proper use and care of opioids. The player navigates through the levels, each covering different opioid safety topics. Level 1, A Quiet Sunday Afternoon, is focused on teaching the player about the safe storage of opioids; the player is taught to lock up medication in a cabinet, so their friends do not take them (Figure 1). The second level, Monday Morning Bus Ride, enhances the game story line by adding a real-life scene about being in pain and forgetting there was an important assignment due that day (Figure 2). Level 3, A Persuasive Speech at School, focuses on teaching the player not to take others pain medication and the negative consequences of taking someone else’s medications; even if 2 people are prescribed a pain killer, that does not mean it is the same kind of medication or dose (Figure 3). Level 4, Bus Ride Home, focuses on teaching the player not to share their medication with others, the negative consequences of sharing medications with others, and what Narcan is used for (Figure 4). Level 5, Last Minute Chore, shows the player the correct way to dispose of an opioid medication and why they should not throw medications away or flush them down the toilet (Figure 5). The scenarios in each level were intended to be realistic and relevant to the adolescents’ daily life.

### Procedure

From October to November 2020, a nationally representative (quotas were set for race or ethnicity and gender based on the US census data) sample of adolescents was recruited via Qualtrics research panels. Parents of eligible adolescents were targeted. Eligible participants were adolescents aged 12 to 18 years who lived in the United States and could speak and understand English. After screening for eligibility, the parents were provided with a link to the online consent document and asked to provide consent for their child to participate in the pregame survey. The child was also provided with an online assent document and asked to provide assent. The parents were then directed to the pregame survey for their child to complete independently. Upon completion of the pregame survey, the parents of participants received compensation based on their predetermined agreement with Qualtrics. The parents were then provided with a link to the online consent document and asked to consent to their child’s participation in gameplay and a postgame survey. The children were also provided with a link to an online assent document and asked to provide assent for participation. Once consent and assent were confirmed, the parents received a link with instructions for their child to play the SG for 30 minutes and complete a postgame survey. Upon verification of gameplay and completion of the postgame survey, a US $10 amazon e-gift card was emailed to the parent of the adolescent for participation. All consent and assent documents detailed the study activities, time commitment, purpose of study, principal investigator’s information, and confidentiality and data security measures.

### Data Analysis

Survey questions were divided into 10 categories to represent key concepts and summarized into concept scores. Pregame and postgame surveys had Likert scores on a 1 through 5 scale and knowledge scores on a 0 to 100% scale; changes in concept scores, from presurvey to postsurvey, were calculated for each participant and were the primary efficacy outcomes. Concept score changes were described by overall mean (SD) and were stratified by gender, race, school, grade, and age. Differences in concept score changes were analyzed using the Kruskal-Wallis tests for categorical characteristics and correlation tests for continuous characteristics. Primary analysis included participants who accessed the game based on IP (located within the United States) and satisfied attention checks for both pregame and postgame surveys. Secondary analyses aimed to (1) assess the differences between participants included in primary analysis vs those who did not meet full attention criteria; and (2) assess the associations between the levels played and the length of play with concept scores. The participants who did not meet full attention criteria either did not access the game or did not self-report playing the game but met other attention criteria (ie, select “A great deal” when asked). No *P* value adjustments were made to account for inflated type 1 error rate. Significance was assessed at the α=.05 level. All statistical analyses* *were performed using R* *statistical software, version 4.0.5 (The R Foundation).

## Results

There were 117 participants who met full attention criteria on pregame and postgame surveys and accessed the game. Of these participants, 55.4% (n=59) identified as male, 48.72% (n=57) identified as White, and the mean age was 14.62 (SD 1.62). The characteristics are shown in Table 1.

**Table 1:** Participant demographic characteristics.

Characteristics		Values
**Grade, n (%)**		
	7	20 (17)
	8	19 (16)
	9	23 (20)
	10	21 (18)
	11	22 (19)
	12	12 (10)
Age (years), mean (SD)		14.62 (1.62)
**Gender,^a^ n (%)**		
	Female	57 (48.7)
	Male	59 (50.4)
	Nonbinary	1 (0.9)
**Race or ethnicity,^b^ n (%)**		
	White or Caucasian	57 (48.72)
	Black or African American	23 (19.66)
	Hispanic or Latinx	6 (5.13)
	Asian	6 (5.13)
	American Indian or Alaskan Native	1 (0.85)
	Native Hawaiian or other Pacific Islander	0 (0)
	More than one selected	24 (20.51)
	Other, please specify	0 (0)

^a^Three options were presented to the participants to select for their gender: “Male,” “Female,” and “Other.”

^b^If participants only selected one category, that was their defined race; all other combinations of selections were defined as “Other.”

These participants significantly improved on all concept scores except for Narcan knowledge (mean change -0.04, SD 0.29; *P*=.60) and safe storage (mean change 0.03, SD 0.20; *P*=.09) (Table 2). Female participants had greater improvement than males for safe disposal (female mean 0.12, SD 0.25 vs male mean 0.04, SD 0.17; *P*=.05) but not for misuse behavior (female mean 0.05, SD 0.28 vs male mean 0.14, SD 0.27; *P*=.04). Perceived knowledge for participants who did not identify as White or were Hispanic had greater improvements than White participants (non-White mean 1.10, SD 1.06 vs White mean 0.75, SD 0.91; *P*=.03). Older grades were associated with greater improvements in opioid knowledge (correlation coefficient -0.23, 95% CI -0.40 to -0.05; *P*=.01). No other associations were significant.

**Table 2:** Concept scores among participants.

Characteristics		Self-efficacy: MUSE^a^	Self-efficacy: opioid safety	Perceived knowledge	Misuse harm	Behavioral intent	Safe storage	Safe disposal	Opioid knowledge	Narcan knowledge	Misuse behavior
**Overall**											
	N	117	117	117	117	117	117	117	117	45	117
	Mean (SD)	0.28 (0.60)	0.39 (0.71)	0.93 (1.00)	0.22 (0.94)	0.25 (0.62)	0.03 (0.20)	0.08 (0.22)	0.06 (0.16)	-0.04 (0.29)	0.09 (0.28)
	Kruskal-Wallis *P* value	<.001	<.001	<.001	.01	<.001	.09	<.001	<.001	.6	<.001
**Gender**											
	Female, mean (SD)	0.35 (0.67)	0.37 (0.70)	1.14 (0.97)	0.15 (0.72)	0.33 (0.59)	0.02 (0.22)	0.12 (0.25)	0.06 (0.16)	0.00 (0.35)	0.05 (0.28)
	Male, mean (SD)	0.21 (0.51)	0.39 (0.74)	0.74 (1.00)	0.27 (1.11)	0.19 (0.65)	0.04 (0.17)	0.04 (0.17)	0.05 (0.16)	-0.06 (0.25)	0.14 (0.27)
	Kruskal-Wallis *P* value	.42	.99	.07	.37	.33	.21	.05	.88	.32	.04
**Race (grouping 1)**											
	White or Caucasian, mean (SD)	0.23 (0.41)	0.30 (0.65)	0.75 (0.91)	0.20 (0.84)	0.23 (0.66)	0.02 (0.14)	0.05 (0.18)	0.03 (0.13)	-0.10 (0.30)	0.10 (0.27)
	Black or African American, mean (SD)	0.27 (0.75)	0.20 (0.80)	0.76 (0.90)	0.15 (1.02)	0.12 (0.65)	0.02 (0.24)	0.12 (0.26)	0.07 (0.22)	0.15 (0.24)	0.01 (0.18)
	Hispanic or Latinx, mean (SD)	0.25 (0.61)	0.58 (0.82)	1.27 (1.22)	0.29 (1.08)	0.40 (0.59)	0.11 (0.29)	0.10 (0.19)	0.09 (0.15)	-0.11 (0.17)	0.18 (0.34)
	Other or missing, mean (SD)	0.54 (0.89)	0.71 (0.45)	1.37 (0.95)	0.30 (1.00)	0.30 (0.47)	-0.02 (0.07)	0.10 (0.32)	0.12 (0.18)	0.17 (0.24)	0.05 (0.33)
	Kruskal-Wallis *P* value	.76	.04	.02	.78	.72	.36	.58	.23	.04	.38
**Race (grouping 2)^b^**											
	White or Caucasian, mean (SD)	0.23 (0.41)	0.30 (0.65)	0.75 (0.91)	0.20 (0.84)	0.23 (0.66)	0.02 (0.14)	0.05 (0.18)	0.03 (0.13)	-0.10 (0.30)	0.10 (0.27)
	Non-White, mean (SD)	0.33 (0.73)	0.47 (0.76)	1.10 (1.06)	0.24 (1.03)	0.27 (0.59)	0.05 (0.24)	0.11 (0.25)	0.09 (0.18)	0.06 (0.24)	0.09 (0.29)
	Kruskal-Wallis *P* value	.73	.21	.03	.42	.63	.73	.23	.26	.12	.80
**School**											
	High school, mean (SD)	0.26 (0.56)	0.34 (0.68)	0.86 (0.94)	0.19 (0.80)	0.23 (0.68)	0.03 (0.21)	0.06 (0.19)	0.04 (0.14)	-0.06 (0.30)	0.12 (0.27)
	Middle school, mean (SD)	0.31 (0.67)	0.48 (0.78)	1.07 (1.11)	0.27 (1.17)	0.30 (0.49)	0.03 (0.17)	0.13 (0.26)	0.10 (0.19)	0.03 (0.25)	0.03 (0.28)
	Kruskal-Wallis *P* value	.42	.21	.23	.51	.45	.77	.06	.34	.39	.15
**Grade**											
	Correlation coefficient	-0.17	-0.14	-0.17	-0.08	-0.09	0	-0.18	-0.23	-0.1	0.12
	95% CI	-0.34 to 0.02	-0.31 to 0.05	-0.34 to 0.02	-0.25 to 0.11	-0.26 to 0.10	-0.18 to 0.18	-0.35 to 0.00	-0.40 to -0.05	-0.38 to 0.20	-0.06 to 0.30
	Pearson's correlation *P* value	.07	.15	.07	.41	.35	.98	.06	.01	.52	.18
**Age**											
	Correlation coefficient	-0.15	-0.12	-0.16	-0.07	-0.06	0	-0.15	-0.18	-0.15	0.1
	95% CI	-0.32 to 0.03	-0.29 to 0.07	-0.33 to 0.03	-0.25 to 0.11	-0.24 to 0.12	-0.18 to 0.19	-0.32 to 0.03	-0.35 to 0.00	-0.42 to 0.15	-0.08 to 0.28
	Pearson's correlation *P* value	.11	.22	.09	.44	.52	.96	.11	.05	.34	.27

^a^MUSE: Medication Understanding and Use Self-Efficacy.

^b^Race grouping 2 examines participants who selected “White” as one category and every other option as “non-White.”

Of the 117 participants who played the game, 28 (23.9%) played all 5 levels. The participants who partially completed the game played an average of 2.42 (SD 1.18) levels (Table 3). Compared with the participants who partially completed the game, completers had a worse change in Narcan knowledge (mean -0.10, SD 0.16) than noncompleters (mean 0.05, SD 0.21; *P*=.04) but better improvement in self-efficacy: opioid safety (completers mean 0.74, SD 0.73 vs noncompleters mean 0.30, SD 0.73; *P*=.01). Completers also had a longer length of play (completers mean 26.94, SD 8.16 minutes vs noncompleters mean 17.41, SD 13.16 minutes; *P*<.001).

**Table 3:** Examining the differences between the participants who played all 5 levels of the game and those who did not.

Variables		Noncompletes	Played all 5 levels	*P* value	
n		67	28	—^a^	
Behavioral intent, mean (SD)		0.24 (0.61)	0.26 (0.54)	.88	
Misuse behavior, mean (SD)		0.10 (0.28)	0.11 (0.28)	.39	
Misuse behavior 2, mean (SD)^b^		0.10 (0.35)	0.04 (0.19)	.32	
Misuse harm, mean (SD)		0.22 (1.04)	0.21 (0.78)	.90	
Narcan knowledge, mean (SD)		0.05 (0.21)	-0.10 (0.16)	.04	
Opioid knowledge: harming teens, mean (SD)^c^		0.09 (0.42)	0.21 (0.42)	.20	
Opioid knowledge, mean (SD)		0.07 (0.15)	0.05 (0.16)	.68	
Perceived knowledge, mean (SD)		0.84 (1.00)	1.04 (0.98)	.43	
Safe disposal, mean (SD)		0.10 (0.22)	0.06 (0.15)	.93	
Safe storage, mean (SD)		0.02 (0.13)	0.03 (0.20)	.66	
Self-efficacy: opioid safety, mean (SD)		0.30 (0.73)	0.74 (0.73)	.01	
Self-efficacy: MUSE,^d^ mean (SD)		0.26 (0.55)	0.35 (0.66)	.58	
Length of play, mean (SD)		17.41 (13.16)	26.94 (8.16)	<.001	
Number of levels played, mean (SD)		2.42 (1.18)	—	—	
**Gender, n (%)**					.32
	Female	36 (53.7)	11 (39.3)	—	
	Male	30 (44.8)	17 (60.7)	—	
	Nonbinary	1 (1.5)	0 (0.0)	—	
Age, mean (SD)		14.36 (1.60)	14.54 (1.62)	.68	
**Race (grouping 1), n (%)**					.14
	A: White or Caucasian	29 (43.3)	13 (46.4)	—	
	B: Black or African American	17 (25.4)	4 (14.3)	—	
	C: Hispanic or Latinx	15 (22.4)	4 (14.3)	—	
	D: Other or missing	6 (9.0)	7 (25.0)	—	
Race (grouping 2) = B: non-White, n (%)^e^		38 (56.7)	15 (53.6)	.78	

^a^Not applicable.

^b^Individual question asking, “Is it okay to take someone else's opioid medication if you have had the same prescription in the past?”

^c^Individual question asking, “Is the opioid crisis harming teenagers in the United States?”

^d^MUSE: Medication Understanding and Use Self-Efficacy.

^e^Race grouping 2 examines participants who selected “White” as one category and every other option as “non-White.”

There were 39 (33%) participants who met full attention criteria on the pregame and postgame surveys but either did not access the game or reported playing the wrong game in the postgame survey. Compared to those who played the game, the participants who did not meet the criteria had worse changes in perceived knowledge (*P*=.05); self-efficacy: opioid safety (*P*=.05); self-efficacy: MUSE (*P*=.01); and when asked the question, “Is it okay to take someone else's opioid medication if you have had the same prescription in the past?” (*P*=.01) (Table 4). Additionally, those who attempted the game but reported playing the wrong game had lower length of play (poor attention mean 7.02, SD 10.23 minutes vs full attention with game mean 20.22, SD 12.64; *P*=.04) and played fewer levels (poor attention mean 1, no SD vs full attention with game mean 3.18, SD 1.54; *P*=.01).

**Table 4:** Examining the differences among participants who accessed the game and had good attention vs those who did not access the game and had poor attention.

Variables		Poor attention, did not access game	Good attention, accessed game	*P* value	
n		39	117	—^a^	
Behavioral intent, mean (SD)		0.17 (0.75)	0.25 (0.62)	.25	
Misuse behavior, mean (SD)		0.05 (0.31)	0.09 (0.28)	.39	
Misuse behavior 2, mean (SD)^b^		-0.08 (0.35)	0.09 (0.32)	.01	
Misuse harm, mean (SD)		-0.04 (0.82)	0.22 (0.94)	.10	
Narcan knowledge, mean (SD)		0.05 (0.28)	-0.04 (0.29)	.44	
Opioid knowledge: harming teens, mean (SD)^c^		0.13 (0.41)	0.13 (0.41)	.99	
Opioid knowledge, mean (SD)		0.09 (0.21)	0.06 (0.16)	.87	
Perceived knowledge, mean (SD)		0.52 (1.13)	0.93 (1.00)	.05	
Safe disposal, mean (SD)		0.09 (0.22)	0.08 (0.22)	.95	
Safe storage, mean (SD)		0.05 (0.15)	0.03 (0.20)	.16	
Self-efficacy: opioid safety, mean (SD)		0.14 (0.71)	0.39 (0.71)	.05	
Self-efficacy: MUSE,^d^ mean (SD)		-0.03 (0.81)	0.28 (0.60)	.01	
Length of play, mean (SD)		7.02 (10.23)	20.22 (12.64)	.04	
Number of levels played, mean (SD)		1.00 (0.00)	3.18 (1.54)	.01	
**Gender, n (%)**					.42
	Female	15 (38.5)	57 (48.7)	—	
	Male	23 (59.0)	59 (50.4)	—	
	Nonbinary	1 (2.6)	1 (0.9)	—	
Age, mean (SD)		14.90 (1.48)	14.62 (1.62)	.32	
**Race (grouping 1), n (%)**					.07
	A: White or Caucasian	26 (66.7)	57 (48.7)	—	
	B: Black or African American	8 (20.5)	23 (19.7)	—	
	C: Hispanic or Latinx	5 (12.8)	23 (19.7)	—	
	D: Other or missing	0 (0.0)	14 (12.0)	—	
Race (grouping 2) = B: non-White, n (%)^e^		13 (33.3)	60 (51.3)	.05	

^a^Not applicable.

^b^Individual questions asking, “Is it okay to take someone else's opioid medication if you have had the same prescription in the past?”

^c^Individual questions asking, “Is the opioid crisis harming teenagers in the United States?”

^d^MUSE: Medication Understanding and Use Self-Efficacy.

^e^Race grouping 2 examines participants who selected “White” as one category and every other option as “non-White.”

There were 13 (11%) participants who failed level 3 but succeeded in level 4, indicating they learned not to share medications with others. These players had longer length of play (mean 27.11, SD 9.56), played more levels (mean 4.38, SD 0.87), and displayed more opioid misuse behaviors during gameplay (mean 3.77, SD 1.88) than all other players (Table 5).

**Table 5:** Players who learned not to share meds vs players with all other patterns.

Variables		All other patterns	Failure in level 3, success in level 4	*P* value	
n		104	13	—^a^	
Behavioral intent, mean (SD)		0.27 (0.64)	0.13 (0.42)	.38	
Misuse behavior, mean (SD)		0.10 (0.29)	0.03 (0.21)	.69	
Misuse behavior 2, mean (SD)^b^		0.10 (0.33)	0.08 (0.28)	.83	
Misuse harm, mean (SD)		0.18 (0.84)	0.55 (1.50)	.13	
Narcan knowledge, mean (SD)		-0.03 (0.30)	-0.08 (0.17)	.47	
Opioid knowledge: harming teens, mean (SD)^c^		0.12 (0.41)	0.15 (0.38)	.83	
Opioid knowledge, mean (SD)		0.05 (0.14)	0.14 (0.25)	.25	
Perceived knowledge, mean (SD)		0.91 (1.00)	1.08 (1.04)	.64	
Safe disposal, mean (SD)		0.08 (0.21)	0.12 (0.26)	.35	
Safe storage, mean (SD)		0.04 (0.20)	0.00 (0.10)	.72	
Self-efficacy: opioid safety, mean (SD)		0.34 (0.70)	0.73 (0.74)	.05	
Self-efficacy: MUSE,^d^ mean (SD)		0.27 (0.56)	0.33 (0.84)	.89	
Length of play, mean (SD)		19.13 (12.77)	27.11 (9.55)	.02	
Number of levels played, mean (SD)		2.99 (1.54)	4.38 (0.87)	.002	
Opioid failures, mean (SD)^e^		2.38 (2.36)	3.77 (1.88)	.03	
**Gender, n (%)**					.88
	Female	50 (48.1)	7 (53.8)	—	
	Male	53 (51.0)	6 (46.2)	—	
	Nonbinary	1 (1.0)	0 (0.0)	—	
Age, mean (SD)		14.69 (1.62)	14.08 (1.55)	.19	
**Race (grouping 1), n (%)**					.54
	A: White or Caucasian	51 (49.0)	6 (46.2)	—	
	B: Black or African American	19 (18.3)	4 (30.8)	—	
	C: Hispanic or Latinx	22 (21.2)	1 (7.7)	—	
	D: Other or missing	12 (11.5)	2 (15.4)	—	
Race (grouping 2) = B: non-White, n (%)^f^		53 (51.0)	7 (53.8)	.84	

^a^Not applicable.

^b^Individual question asking, “Is it okay to take someone else's opioid medication if you have had the same prescription in the past?”

^c^Individual question asking, “Is the opioid crisis harming teenagers in the United States?”

^d^MUSE: Medication Understanding and Use Self-Efficacy.

^e^Players failed the level due to an opioid misuse behavior (failure to lock up opioids, sharing opioids, etc) vs a non–opioid-related failure (failure to get a hall pass, forgetting notes, etc).

^f^Race grouping 2 examines participants who selected “White” as one category and every other option as “non-White.”

## Discussion

### Principal Findings

This paper describes the use of an SG designed to educate adolescents on opioid safety. The game was evaluated in terms of impact on the players’ pregame and post survey results for self-efficacy, perceived knowledge, misuse harm and behavior, behavioral intent, safe storage and disposal, opioid knowledge, and Narcan knowledge. The results indicate significant improvement in all areas except for Narcan knowledge and safe storage. However, not all remaining improvements were still significant when compared to a self-selecting control group of participants who either did not play the game or reported playing the wrong game. Those who accessed the game had better improvement in misuse behavior, perceived knowledge, and self-efficacy. Thus, the findings from this study suggest the potential effectiveness of the SG, MedSMART: Adventures in PharmaCity on improving opioid medication safety awareness among adolescents.

The findings from this study revealed female participants had better improvement for safe disposal, but males had greater improvement for misuse behavior. The participants who were non-White or Hispanic had higher improvements in perceived knowledge than White participants, and older participants were associated with greater improvements in opioid knowledge. There are limited studies that examine why these differences exist amongst gender, race, and age [31,32]. Those that examine these differences typically only study adults and may not apply a pre-post survey study design [33]. Racial and ethnic disparities in pain management are well documented in literature and may contribute to these differences as well [34-38]. Studies show that not only are non-White patients less likely to receive opioid prescriptions for certain conditions, but also that disparities exist in access to specialized care, such as mental health, addiction, or pain specialists [34-38]. Data from this study and current literature suggest that research focused on these aspects is warranted in the adolescent population.

### Implications

Studies have indicated that SGs possess the potential for increasing students’ learning, motivation, and engagement as well as the ability to develop their minds and improve their learning efficiency [39-41]. Additionally, the Pediatric Pharmacy Advocacy Group recommends pharmacists educate adolescents about the proper administration, storage, and disposal of opioids [42]. Current literature suggests that reducing opioid prescribing by health care professionals, specifically dentists and surgeons, could substantially lower prescription opioid exposure in adolescents [11]. There is a need for initiatives that target high-volume prescribers and provide medication safety education for the patients.

An opportunity exists to implement MedSMART: Adventures in PharmaCity in multiple settings: (1) a school setting where students can play the game in a health course or a similar setting; (2) a doctor’s office where the adolescent is being prescribed the opioid and could play the game in the waiting room; and (3) at the pharmacy where the adolescent is picking up their opioid prescription. The addition of an SG in any of these settings may help proactively curb opioid misuse in adolescents.

### Limitations

This single-arm study did not have a randomized researcher-blinded control group. Although the participants who did not play the game or who reported playing the wrong game were compared to those who did play the game (Table 4), this was a self-selecting control group and not randomized. Efficacy outcomes relied on self-completion questionnaires, which may have been affected by participant dishonesty and reporting bias.

Additionally, although the sample demographics were similar to those of the US demographics, the majority of the participants were White. Thus, generalizability of study findings may be somewhat limited, as there are racial and ethnic differences in access to prescription opioids and pain management.

The participants could not ask for assistance navigating the game or clarification on survey questions because they were not monitored by research staff during data collection. Moreover, the participants’ attentiveness during gameplay was not observed. The study design relied on trusting the parent to not influence their child’s survey responses or gameplay. To ensure accuracy, future designs should confirm that adolescents are taking the surveys and playing the game and that parents are not affecting the responses. A focus group or interview could also be incorporated to a study’s design to understand what players learned from the game or to identify potential game improvements.

### Conclusion

Health messaging surrounding opioid safety requires novel and engaging strategies to be effective in increasing knowledge, changing behavior, and preventing prescription opioid misuse. The SG MedSMART: Adventures in PharmaCity was designed to engage adolescents in real-life scenarios and provide them with the information needed to correctly use opioids. The findings from this study show that the SG significantly improved all concept scores except for Narcan knowledge and safe storage. Future research should examine how the game can improve adolescents’ understanding for safe opioid storage and the use of Narcan. MedSMART: Adventures in PharmaCity could be a useful and effective tool for preventive opioid misuse intervention programs and should replicate this study using a randomized controlled trial with a prepost survey study design. Further research is needed to assess potential benefits of dissemination and implementation of the game in health care or school settings.

## Appendix

Multimedia Appendix 1 Survey questions, composite scores, and response types in the pregame survey.

Multimedia Appendix 2 Survey questions, composite scores, and response types in the postgame survey.

## Abbreviations

MUSE: Medication Understanding and Use Self-Efficacy
SG: serious game
