# Supplementary material for: Investigating the Use of a Serious Game to Improve Opioid Safety Awareness Among Adolescents: Quantitative Study
Source: JMIR Serious Games. 2021 Dec 23;9(4):e33975. doi: 10.2196/33975 (PMC8738992; doi:10.2196/33975)
Supplement: Multimedia Appendix 1 [file games_v9i4e33975_app1.docx]

Multimedia Appendix 1**.** Survey questions, composite scores, and response types in the pre-game survey.

| **Category** | **Question** | **Response Type** |  |
| --- | --- | --- | --- |
| Opioid knowledge | Can opioid medications make you dizzy or sleepy even when they are taken as prescribed by your doctor? | Yes; No; Don’t know |  |
|  | If you do not know how much of an opioid medication to take, is it ok to ask your friends? |  |  |
|  | Can taking too much of an opioid medication cause you to pass out? |  |  |
|  | Is it safe to drive a car or supervise children after you have taken your prescribed amount of opioid medication? |  |  |
|  | Is constipation a sign of opioid medication dependence or addiction? |  |  |
|  | Can opioid medications cause harm when not used as prescribed by your doctor? |  |  |
|  | Can extra opioid medications be shared with your friends if they are in pain? |  |  |
|  | If you take an opioid medication correctly, can there still be side effects? |  |  |
|  | Is the opioid crisis harming teenagers in the U.S.? * |  |  |
|  |  |  |  |
| Safe storage | Should prescription opioids be stored… in the medicine cabinet? |  |  |
|  | Should prescription opioids be stored… in an unlocked drawer or cabinet? |  |  |
|  | Should prescription opioids be stored… in a purse or handbag? |  |  |
|  | Should prescription opioids be stored… in a locked place, such as a lock box, safe, or locked drawer? |  |  |
|  |  |  |  |
| Safe disposal | Should you get rid of unused prescription opioids by… throwing them in the trash? |  |  |
|  | Should you get rid of unused prescription opioids by… dropping them off in a dispoal box? |  |  |
|  | Should you get rid of unused prescription opioids by… flushing them down the toilet? |  |  |
|  | Should you get rid of unused prescription opioids by… putting them in cat litter or coffee grinds? |  |  |
|  | Should you get rid of unused prescription opioids by… taking them to a pharmacy, doctor, or hospital? |  |  |
|  | Should you get rid of unused prescription opioids by… putting them down the sink/disposal? |  |  |
|  |  |  |  |
| Perceived knowledge | How much do you know about…how to use an opioid medication safely? | Likert: 1, none; 2, a little; 3, some; 4, quite a bit; 5, a great deal |  |
|  | How much do you know about…what counts as misuse of an opioid medication? |  |  |
|  | How much do you know about…the harmful effects of misusing opioids? |  |  |
|  | How much do you know about…how to store opioids safely? |  |  |
|  | How much do you know about…what you should do in situations involving an opioid overdose? |  |  |
|  | How much do you know about…how to dispose of opioids safely? |  |  |
|  |  |  |  |
| Self-efficacy: MUSE | It is easy for me to ask my parent questions about safe opioid use. | Agreement scale: 1, strongly disagree; 2, slightly disagree; 3, neutral; 4, slightly agree; 5, strongly agree |  |
|  | It is easy for me to understand my parent's instructions for using opioids safely. |  |  |
|  | It is easy for me to understand instructions on how to safely manage opioids. |  |  |
|  | It is easy for me to get all the information I need about safe opioid use. |  |  |
|  |  |  |  |
| Self-efficacy: opioid safety | How confident are you that you can... | Confidence scale 1, not at all confident; 2, slightly; 3, somewhat; 4, very; 5, extremely confident |  |
|  | use opioid medication as directed? |  |  |
|  | know where your medication is at all times? |  |  |
|  | store your medication in a locked area? |  |  |
|  | dispose of your medication in a dropbox? |  |  |
|  | tell a friend no if they ask to share your medication? |  |  |
|  | only take medication that was prescribed for you? |  |  |
|  | encourage others to use opioids safely? |  |  |
|  |  |  |  |
| Misuse harm | How much harm does misuse of opioids do to a person's…physical health? | Likert: 1, none; 2, a little; 3, some; 4, quite a bit; 5, a great deal |  |
|  | How much harm does misuse of opioids do to a person's…mental health? |  |  |
|  | How much harm does misuse of opioids do to a person's…ability to do well in school? |  |  |
|  | How much harm does misuse of opioids do to a person's…relationships with their family? |  |  |
|  | How much harm does misuse of opioids do to a person's…relationships with their peers or friends? |  |  |
|  |  |  |  |
| Misuse behavior | Is someone misusing opioids if… they return their unused opioid medication to the pharmacy when it expires? | Yes; No; Don't know |  |
|  | Is someone misusing opioids if… they use their prescribed opioid after it expires? |  |  |
|  | Is someone misusing opioids if… they use someone else's opioid medication? |  |  |
|  | Is someone misusing opioids if… they use opioids more often than their prescription calls for? |  |  |
|  | Is someone misusing opioids if… they share their opioid medications with others? |  |  |
|  | Is someone misusing opioids if… they take their opioid medication for a reason different than what it was prescribed for? |  |  |
|  | Is it okay to take someone else's opioid medication if you have had the same prescription in the past?* |  |  |
|  |  |  |  |
| Narcan® knowledge | Have you heard of the drug naloxone (Narcan®)? | Yes; No,  If ‘Yes’ to first question, then ‘Yes; No; Don't know’ to additional questions. |  |
|  | (if yes) Is Naloxone (Narcan®) used to… reverse only heroin overdoses? |  |  |
|  | (if yes) Is Naloxone (Narcan®) used to… help heroin users detox? |  |  |
|  | (if yes) Is Naloxone (Narcan®) used to… reverse any opioid overdose? |  |  |
|  |  |  |  |
| Behavioral intent | How likely are you to do the following? | Likelihood scale: 1, not at all; 2, slightly; 3, somewhat; 4, very; 5, extremely likely |  |
|  | use opioid medication as directed? |  |  |
|  | know where my medication is at all times? |  |  |
|  | store my medication in a locked area? |  |  |
|  | dispose of my medication in a dropbox? |  |  |
|  | share my medication with a friend in need? |  |  |
|  | take medication that was prescribed for someone else? |  |  |
|  | encourage others to use opioids safely? |  |  |
|  |  |  |  |
| Perceived game effect | After playing an educational video game, would you feel as though your knowledge about opioid medication safety would increase? | Yes; No |  |
|  |  |  |  |
| Attention check | Please select "a great deal." This question is to make sure you are still paying attention. | As directed in the question |  |
|  | This is to make sure you are still paying attention. Please select "somewhat." |  |  |
|  |  |  |  |
| Demographics | Year in school | 7 - 12, number = grade |  |
|  | Gender | 1, Female; 2, Male; 3, Transgender; 4, Nonbinary; 5, Other |  |
|  | Age | 12 – 18 years old |  |
|  | How many kids under 18 live in your household? Do not count yourself. | Insert number |  |
|  | Race/ethnicity | 1, American Indian or Alaskan Native; 2, Asian; 3, Black or African American; 4, Hispanic or Latinx; 5, Native Hawaiian or Other Pacific Islander; 6, White or Caucasian; 7, Other: Please specify |  |
|  |  |  |  |
|  |  |  |  |
|  |  |  |  |
|  |  |  |  |
|  |  |  |  |
|  |  |  |  |
|  |  |  |  |
|  |  |  |  |
|  |  |  |  |

*Question was analyzed individually as well.
