# Supplementary material for: Investigating the Use of a Serious Game to Improve Opioid Safety Awareness Among Adolescents: Quantitative Study
Source: JMIR Serious Games. 2021 Dec 23;9(4):e33975. doi: 10.2196/33975 (PMC8738992; doi:10.2196/33975)
Supplement: Multimedia Appendix 2 [file games_v9i4e33975_app2.docx]

Multimedia Appendix 2**.** Survey questions, composite scores, and response types in the post-game survey.

| **Category** | **Question** | **Response Type** |
| --- | --- | --- |
| Attention | Which of the following games did you play? | 1, Eat Right Cafe; 2, Cancer Fighter; 3, MedSmart: Adventures in Pharmacity |
|  |  |  |
| Engagement/Preference | In two sentences, please describe the game you played in your own words. | Free Response |
|  |  |  |
| Game experience | How enjoyable was playing the game? | 1, Not at all; 2, slightly; 3, somewhat; 4, very; 5, extremely |
|  | How interested are you in playing this game again? |  |
|  | How likely would you be to recommend this game to a friend or family member? |  |
|  | How willing were you to continue playing? |  |
|  | How clear was it you what you should do next in the game? |  |
|  | How realistic did you find how the game showed safe use of opioids? |  |
|  | How bored were you while playing this game? |  |
|  | How easy was this game to play? |  |
|  |  |  |
| Informative aspect | What aspects about opioid safety did the game best inform you about? (check all that apply) | 1, safe disposal; 2, proper use; 3, safe storage; 4, overdose; 5, harmful effects; 6, other |
|  |  |  |
| Like best | What did you like best about this game? (check all that apply) | 1, graphics; 2, characters; 3, topic; 4, storyline; 5, challenges; 6, other |
|  |  |  |
| Scenario evaluation | Which scenario in the game was… most fun to play | 1, level 1 home; 2, level 3 school; 3, level 4 bus; 4, level 5 disposal; 5, no level |
|  | Which scenario in the game was… most interesting |  |
|  | Which scenario in the game was… most difficult to play |  |
|  | Which scenario in the game was… most informative (e.g. taught you the most) |  |
|  |  |  |
| Assessing game design/mechanics | What could have been done better in designing this game? | Free response |
|  |  |  |
| Game evaluation | Overall, this is a well-designed game. | Agreement scale: 1, strongly disagree; 2, slightly disagree; 3, neutral; 4, slightly agree; 5, strongly agree |
|  | The game is user-friendly. |  |
|  | The information in this game is reliable. |  |
|  | The information is personally relevant to me. |  |
|  | I learned a lot from this game. |  |
|  |  |  |
| Where to play | Where would you be most interested in playing this game? Check all that apply | 1, school; 2, home; 3, doctor; 4, clinic; 5, hospital; 6, pharmacy; 7, other location |
|  |  |  |
| Prior experience | Have you ever been prescribed an opioid by a doctor before? | 1, yes; 2, unsure; 3, no; 4, prefer not to answer |
|  |  |  |
| Prescribed opioid | Please list the opioids you have been prescribed | text |
|  |  |  |
| Possible opioid prescription | Please list medications you have been prescribed that you think may be opioids | text |
|  |  |  |
| Opioid knowledge | Can opioid medications make you dizzy or sleepy even when they are taken as prescribed by your doctor? | Yes; No; Don’t know |
|  | If you do not know how much of an opioid medication to take, is it ok to ask your friends? |  |
|  | Can taking too much of an opioid medication cause you to pass out? |  |
|  | Is it safe to drive a car or supervise children after you have taken your prescribed amount of opioid medication? |  |
|  | Is constipation a sign of opioid medication dependence or addiction? |  |
|  | Can opioid medications cause harm when not used as prescribed by your doctor? |  |
|  | Can extra opioid medications be shared with your friends if they are in pain? |  |
|  | If you take an opioid medication correctly, can there still be side effects? |  |
|  | Is the opioid crisis harming teenagers in the U.S.? * |  |
|  |  |  |
| Safe storage | Should prescription opioids be stored… in the medicine cabinet? |  |
|  | Should prescription opioids be stored… in an unlocked drawer or cabinet? |  |
|  | Should prescription opioids be stored… in a purse or handbag? |  |
|  | Should prescription opioids be stored… in a locked place, such as a lock box, safe, or locked drawer? |  |
|  |  |  |
| Safe disposal | Should you get rid of unused prescription opioids by… throwing them in the trash? |  |
|  | Should you get rid of unused prescription opioids by… dropping them off in a disposal box? |  |
|  | Should you get rid of unused prescription opioids by… flushing them down the toilet? |  |
|  | Should you get rid of unused prescription opioids by… putting them in cat litter or coffee grinds? |  |
|  | Should you get rid of unused prescription opioids by… taking them to a pharmacy, doctor, or hospital? |  |
|  | Should you get rid of unused prescription opioids by… putting them down the sink/disposal? |  |
|  |  |  |
| Perceived knowledge | How much do you know about…how to use an opioid medication safely? | Likert: 1, none; 2, a little; 3, some; 4, quite a bit; 5, a great deal |
|  | How much do you know about…what counts as misuse of an opioid medication? |  |
|  | How much do you know about…the harmful effects of misusing opioids? |  |
|  | How much do you know about…how to store opioids safely? |  |
|  | How much do you know about…what you should do in situations involving an opioid overdose? |  |
|  | How much do you know about…how to dispose of opioids safely? |  |
|  |  |  |
| Self-efficacy: MUSE | It is easy for me to ask my parent questions about safe opioid use. | Agreement scale: 1, strongly disagree; 2, slightly disagree; 3, neutral; 4, slightly agree; 5, strongly agree |
|  | It is easy for me to understand my parent's instructions for using opioids safely. |  |
|  | It is easy for me to understand instructions on how to safely manage opioids. |  |
|  | It is easy for me to get all the information I need about safe opioid use. |  |
|  |  |  |
| Self-efficacy: opioid safety | How confident are you that you can... | Confidence scale 1, not at all confident; 2, slightly; 3, somewhat; 4, very; 5, extremely confident |
|  | use opioid medication as directed? |  |
|  | know where your medication is at all times? |  |
|  | store your medication in a locked area? |  |
|  | dispose of your medication in a dropbox? |  |
|  | tell a friend no if they ask to share your medication? |  |
|  | only take medication that was prescribed for you? |  |
|  | encourage others to use opioids safely? |  |
|  |  |  |
| Misuse harm | How much harm does misuse of opioids do to a person's…physical health? | Likert: 1, none; 2, a little; 3, some; 4, quite a bit; 5, a great deal |
|  | How much harm does misuse of opioids do to a person's…mental health? |  |
|  | How much harm does misuse of opioids do to a person's…ability to do well in school? |  |
|  | How much harm does misuse of opioids do to a person's…relationships with their family? |  |
|  | How much harm does misuse of opioids do to a person's…relationships with their peers or friends? |  |
|  |  |  |
| Misuse behavior | Is someone misusing opioids if… they return their unused opioid medication to the pharmacy when it expires? | Yes; No; Don't know |
|  | Is someone misusing opioids if… they use their prescribed opioid after it expires? |  |
|  | Is someone misusing opioids if… they use someone else's opioid medication? |  |
|  | Is someone misusing opioids if… they use opioids more often than their prescription calls for? |  |
|  | Is someone misusing opioids if… they share their opioid medications with others? |  |
|  | Is someone misusing opioids if… they take their opioid medication for a reason different than what it was prescribed for? |  |
|  | Is it okay to take someone else's opioid medication if you have had the same prescription in the past?* |  |
|  |  |  |
| Narcan® knowledge | Have you heard of the drug naloxone (Narcan®)? | Yes; No,  If ‘Yes’ to first question, then ‘Yes; No; Don't know’ to additional questions. |
|  | (if yes) Is Naloxone (Narcan®) used to… reverse only heroin overdoses? |  |
|  | (if yes) Is Naloxone (Narcan®) used to… help heroin users detox? |  |
|  | (if yes) Is Naloxone (Narcan®) used to… reverse any opioid overdose? |  |
|  |  |  |
| Behavioral intent | How likely are you to do the following? | Likelihood scale: 1, not at all; 2, slightly; 3, somewhat; 4, very; 5, extremely likely |
|  | use opioid medication as directed? |  |
|  | know where my medication is at all times? |  |
|  | store my medication in a locked area? |  |
|  | dispose of my medication in a dropbox? |  |
|  | share my medication with a friend in need? |  |
|  | take medication that was prescribed for someone else? |  |
|  | encourage others to use opioids safely? |  |
|  |  |  |
| Attention check | Please select "a great deal." This question is to make sure you are still paying attention. | As directed in the question |
|  | This is to make sure you are still paying attention. Please select "somewhat." |  |

*Question was analyzed individually as well.
